# Identification of transcription factor-lipid droplet-related gene biomarkers for the prognosis of post-acute myocardial infarction-induced heart failure

**DOI:** 10.3389/fcvm.2024.1429387

**Published:** 2024-12-12

**Authors:** Jianqiao Zhao, Can Guo, Mengyuan Cheng, Jie Li, Yangyang Liu, Huahua Wang, Jianping Shen

**Affiliations:** Department of Cardiology, Affiliated Hospital of Integrated Traditional Chinese and Western Medicine, Nanjing University of Chinese Medicine, Nanjing, Jiangsu, China

**Keywords:** lipid droplet-related genes, myocardial infarction, heart failure, transcription factor, peripheral blood, ARID3a, ABHD5

## Abstract

**Introduction:**

Patients with acute myocardial infarction (AMI) are at high risk of progressing to heart failure (HF). Recent research has shown that lipid droplet-related genes (LDRGs) play a crucial role in myocardial metabolism following MI, thereby influencing the progression to HF.

**Methods:**

Weighted gene co-expression network analysis (WGCNA) and differential expression gene analysis were used to screen a transcriptome dataset of whole blood cells from AMI patients with (AMI HF, *n* = 16) and without progression (AMI no-HF, *n* = 16). Functional enrichment analysis were performed to observe the involved function. Machine learning methods were used to screen the genes related to prognosis. Transcriptional factors (TF) were predicted by using relevant databases. ROC curves were drawn to evaluate the TF-LDRG pair in predicting HF in the validation dataset (*n* = 16) and the clinical trial (*n* = 13).

**Results:**

The 235 identified genes were primarily involved in pathways related to fatty acid and energy metabolism. 22 genes were screened out that they were strongly associated with prognosis. 35 corresponding transcription factors were predicted. The TF-LDRG pair, ABHD5-ARID3a, was demonstrated good predictive accuracy.

**Discussion:**

Our findings suggest that ABHD5-ARID3a have significant potential as predictive biomarkers for heart failure post-AMI which also provides a foundation for further exploration into the molecular mechanisms underlying the progression from AMI to HF.

## 1 Introduction

With advances in coronary artery interventions and the standardization of early reperfusion strategies, an increasing number of patients with a myocardial infarction survive after acute coronary artery occlusion and reperfusion. However, acute ischemia and reperfusion can cause myocardial injury and ventricular remodeling, and ultimately lead to heart failure (HF), which seriously affects the long-term prognosis of patients (1). The incidence of heart failure in the Chinese population within 7 days after a myocardial infarction is 19.3%, and the incidence of heart failure within 30 days–6.7 years after a myocardial infarction is 13.1%–37.5% (2). To improve the treatment and management of heart failure after an acute myocardial infarction (AMI), the most important step is the rapid and accurate diagnosis of HF after MI. Currently, the diagnosis of HF is mainly based on serum biomarkers such as brain natriuretic peptide (BNP), NT-proBNP, C-reactive protein (CRP), and soluble ST2 (sST2) (3, 4). However, these biomarkers have many limitations because they lack specificity, as they can be elevated in many non-HF conditions, and they also lack predictive utility for HF, potentially resulting in a lack of early interventions for the complication (5). Biomarkers are characterized by their low cost, minimal risk, and rapid detection, and have the potential to provide valuable insight into the complex development of HF after an AMI. Early testing of serum biomarker gene expression holds promise for accurately detecting and monitoring HF after an AMI (6).

Lipid droplets (LD) are the lipid storage organelles in mammalian cells, providing a means for cells to store energy in the form of lipids (7). The surface of lipid droplets is covered with numerous proteins, known as lipid droplet-associated proteins, which can regulate lipid metabolism, thereby potentially influencing cell fate determination. For example, the perilipin (PLIN) family of proteins can regulate lipid hydrolysis and interact with mitochondria to modulate fatty acid β-oxidation to support energy supply (8). The hypoxia-inducible lipid droplet-associated (HILPDA) protein can be activated to increase intracellular lipids in response to stress overload (9). Lipid droplet-attached proteins, such as UCP1, have been reported to promote thermogenesis in HF (10). Cell death-inducing DFFA-like effector (CIDE) proteins, which are considered to be involved in LD–LD fusion, were observed to correlate with the severity of HF (11). The lipid droplet-related genes (LDRGs), which code these proteins, may serve as potential candidate genes for cardiac abnormalities. After an AMI, the myocardium switches from energy utilization via fatty acid β-oxidation to glycolysis. Even after blood flow is restored, intracellular calcium overload and increased oxidative stress exacerbate cell damage, while lipid metabolism remains impaired within the mitochondria (12). This leads to reduced energy production, which is a critical factor in the development of HF after an AMI.

To bridge this gap, we obtained peripheral blood sample data from individuals who had experienced an AMI with or without subsequent HF from the Gene Expression Omnibus (GEO) database. We used machine-learning methods to screen the LDRGs for potential biomarkers for HF after an MI. We also predicted the regulatory relationships of these genes and investigated the transcription factors (TFs) that regulate these genes (13). TFs are crucial factors that influence the expression of LDRGs and jointly exploring TFs and LDRGs can enhance the accuracy of prognostic predictions. The combined gene expression of TFs and LDRGs was rigorously evaluated and validated for the accurate prediction of the occurrence of HF after an AMI through a publicly available online dataset and samples collected from actual clinical practice. Our study focused on lipid droplet-related genes and identified potential biomarkers for HF after an AMI and their regulatory networks. This research may aid in the rapid identification of HF complications after an AMI in clinical settings, allowing for informed clinical decision-making.

## 2 Materials and methods

### 2.1 Data collection

In our study, we obtained two datasets, GSE11947 and GSE59867, from the GEO database. The GSE11947 dataset consists of 32 peripheral blood samples from individuals who had experienced an acute myocardial infarction 12 h earlier. These samples were divided into two groups based on whether or not they developed heart failure at 1 month: the non-heart failure group (*n* = 16) and the heart failure group (*n* = 16). This dataset contains gene expression data for up to approximately 25,000 genes on an oligonucleotide microarray.

In the GSE59867 dataset, the peripheral blood gene expression data on day 1 after the onset of a myocardial infarction was obtained. The occurrence of heart failure was assessed at the 1-month mark, with eight cases developing heart failure and eight cases not developing heart failure.

### 2.2 Screening LDRGs using machine learning

Four standard machine-learning algorithm models [support vector machine (SVM), naive Bayes, classification and regression tree (CART), and random forest] (14) were trained based on the GSE11947 dataset using the caret package. The training process included three iterations of 10-fold validation to obtain the optimal parameter values. The model with the highest accuracy among these algorithms was selected as the final model. Disease-relevant genes were then further filtered from the dataset using this final model.

### 2.3 Differential expression analysis in HF compared with non-HF after a myocardial infarction

In the GSE11947 dataset, gene IDs were converted to symbol format and mRNA expression levels were log2 transformed. The differential expression analysis between the non-HF and HF groups was performed using the “limma” package (15). A cut-off threshold of 1.2 was applied and statistically significant differences were identified based on adjusted *p*-values below 0.05. The results were plotted using a volcano plot to visually show the changes in expression.

### 2.4 Single-sample gene set enrichment analysis

Single-sample gene set enrichment analysis (ssGSEA) is an extension of gene set enrichment analysis used for quantifying biological processes. This method represents the concrete level of gene set enrichment in the biological process within a given dataset (16). In our study, we conducted ssGSEA using the “GSVA” package to investigate the association between LDRGs and post-AMI HF. The LDRGs included the genes mentioned in the previous study (17). An enrichment score was calculated for each sample. The significance of the difference between the scores of the non-HF and HF groups was determined using the *t*-test method.

### 2.5 Weighted gene co-expression network analysis

Weighted gene co-expression network analysis (WGCNA) is a technique used for investigating gene co-expression patterns in a given dataset. In this study, we filtered genes based on their variance, using the GSE11947 dataset, and created a co-expression network with the selected genes. We established soft thresholding to achieve a scale-free topology, which determined the connectivity and relationship strength between the genes. The study employed a topological overlap matrix (TOM) to detect gene modules, which are groups of genes exhibiting a high degree of correlated expression patterns.

Furthermore, our study utilized module eigengenes (MEs), which are representative values for each module. We selected modules that demonstrated noticeable differences in association with the clinical manifestation of HF.

### 2.6 Functional and pathway enrichment analysis

Gene Ontology (GO) and Kyoto Encyclopedia of Genes and Genomes (KEGG) enrichment analysis of the overlaps between the differentially expressed genes and the modules selected by the WGCNA was performed using the Metascape website (https://metascape.org/). Statistical significance was determined for enriched GO terms and pathways based on a threshold of *p*-value <0.05.

### 2.7 Construction of a transcription factor and lipid droplet-related gene network

Transcription factor prediction for lipid droplet-related genes was performed using several databases including ChIP-X enrichment analysis (ChEA), Encyclopedia of DNA elements (ENCODE), human transcription factor target database (hTFtarget), transcription factor database (TRANSFAC), and transcriptional regulatory relationships unraveled by sentence-based text mining (TRRUST) (18–21). Protein–protein interaction (PPI) analysis between the transcription factors and lipid droplet-related genes was also performed using the search tool for the retrieval of interacting genes (STRING) database to unravel potential regulatory relationships.

The overlapping set of predicted transcription factors and differentially expressed genes was obtained and used to construct a heat map illustrating the expression correlation between these genes and lipid droplet-related genes. Statistical significance was determined using a threshold of *p* <0.05 to indicate significant differences.

### 2.8 Evaluation and validation of the TF-LDRG predictive markers

A receiving operator characteristic (ROC) curve was constructed based on the gene expression data from the GSE11947 dataset to evaluate the specificity and sensitivity of different transcription factor-lipid droplet-related genes (TF-LDRGs) in predicting heart failure. The ROC curve was constructed using the pROC package. The occurrence of heart failure was the label and the gene expression data was the predictor. The gene expression value from each sample was used as the cut-off value. The true positive and false positive rates were calculated based on each cut-off value for all samples. The ROC curve was then plotted. For constructing the ROC curve of the gene pairs, logistic fitting was performed to generate predictions within the expression of the pairs of genes, followed by ROC curve plotting. The area under the ROC curve (AUC) was calculated to determine the reliability of the prediction. In addition, the predictive performance of the combination for assessing heart failure was validated using ROC curves generated from the 1st-day gene expression data in the GSE55978 dataset.

### 2.9 Clinical samples

Clinical samples were obtained from patients undergoing emergency coronary angiography and percutaneous coronary intervention (PCI) using peripheral blood collection within the catheterization laboratory from December 2023 to March 2024 in the Jiangsu Province Hospital of Integration of Chinese and Western Medicine. This study was approved by the Ethics Committee of the Jiangsu Province Hospital of Integrated Traditional Chinese and Western Medicine (2023-LWKYZ-077). The diagnostic criteria for ST-segment elevation myocardial infarction (STEMI) are as follows: age between 18 and 75, chest pain, ST-segment elevation, and cardiac biomarkers levels above the normal threshold.

Eligible patients were selected for peripheral blood collection to examine the expression of the identified TF-LDRGs. Patients who returned for a follow-up visit between 2 and 6 weeks after their PCI were clinically diagnosed as either with or without heart failure and were accordingly categorized into either the AMI_HF group or AMI_noHF group.

### 2.10 Peripheral blood mRNA expression analysis

The procedure involves initially centrifuging 1 ml of whole blood at 3,000 rpm for 5 min to remove the supernatant. Red blood cell lysis buffer was then added, followed by vertexing and 10-min incubation. After another centrifugation and supernatant removal, RNA extraction buffer was introduced along with chloroform, and then mixed through inversion. The mixture was chilled at 4℃ and centrifuged at 12,000 rpm. The resulting supernatant (400 µl) was transferred to a new tube, mixed with isopropanol, and centrifuged again under similar conditions. Finally, the supernatant was discarded, and the RNA pellet was resolubilized in 20 µl of nuclease-free water.

RNA was reverse-transcribed into cDNA following the instructions of a commercial reverse transcription kit. Primers, quantitative polymerase chain reaction (qPCR) mix, and nuclease-free water were then added to the cDNA. This was followed by denaturation, annealing, and extension to complete the real-time quantitative PCR.

### 2.11 Statistical analysis

All analyses were performed using *R* software (4.3.1) Spearman’s correlation analysis was utilized to test correlation coefficients. A *p*-value <0.05 combined with a false discovery rate <0.05 was considered a significant difference in all findings of the study. For the baseline data of the patients, Fisher's test was used for the count data, and the *t*-test was used for the quantitative data. In the clinical samples, we used the *t*-test for the analysis of the difference in the expression of a single gene between the two groups.

## 3 Results

### 3.1 Gene functional enrichment between non-HF and HF patients

**Figure 1:**
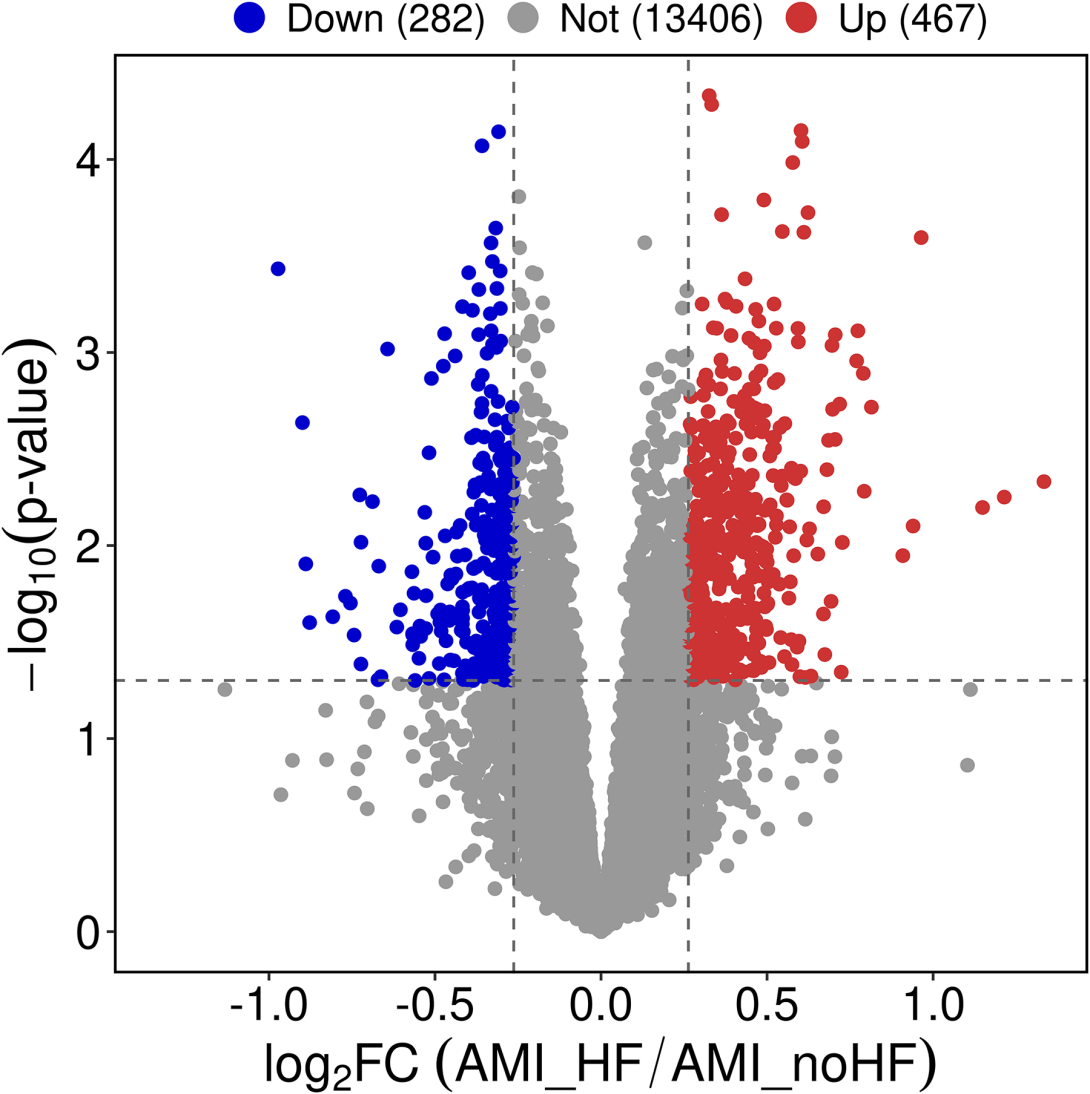
Differential gene expression analysis of peripheral blood samples in patients with and without heart failure after a myocardial infarction.

The expression matrix obtained from GSE11947 consists of 16 non-HF and 16 HF patients, yielding a total of 749 differentially expressed genes. Among these genes, 467 genes were upregulated and 282 genes were downregulated in the heart failure group compared to the non-heart failure group. These differentially expressed genes were visualized using volcano plots (Figure 1).

**Figure 2:**
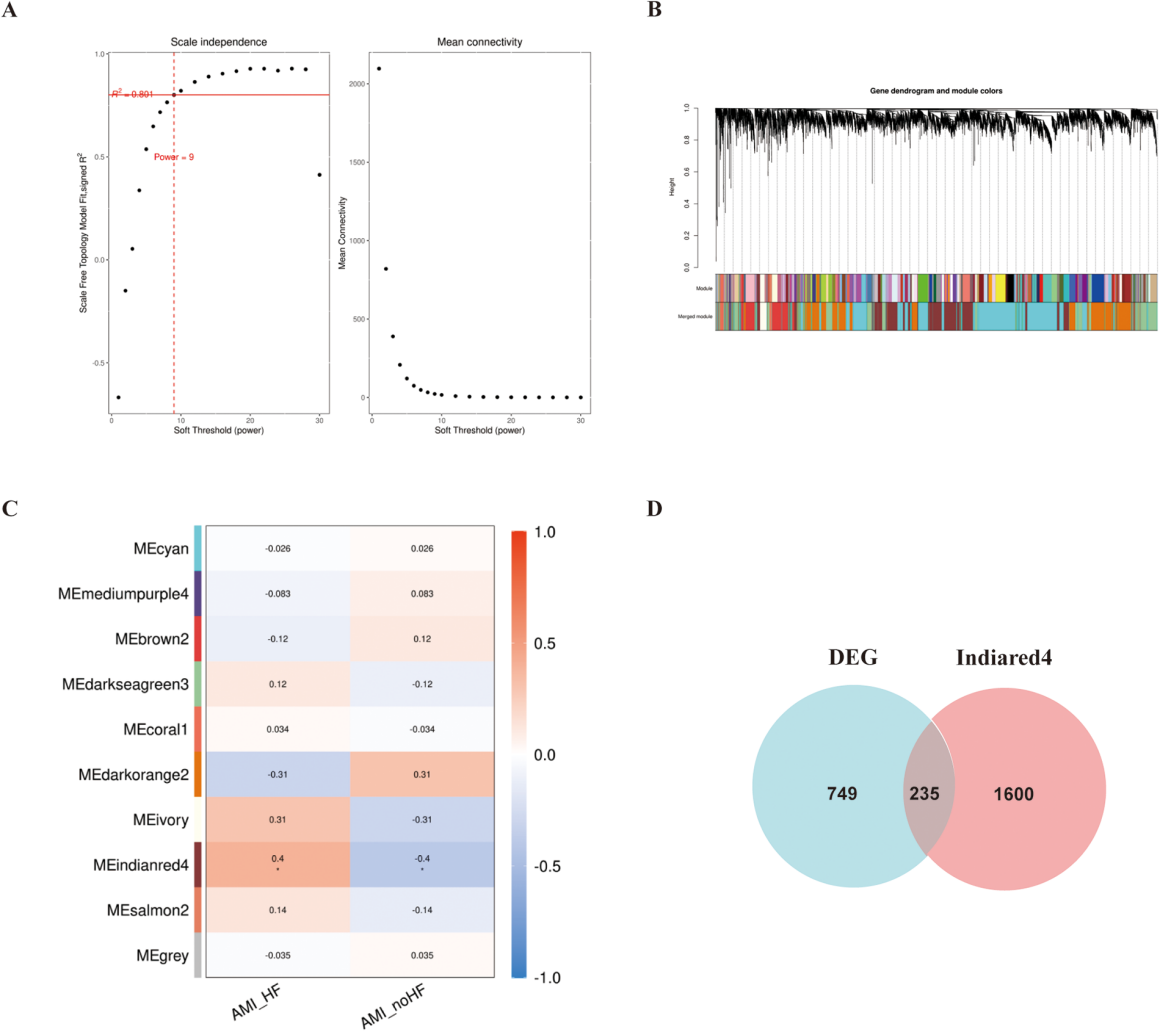
WGCNA analysis of the GSE11947 gene expression matrix. **(A)** Scale-free network model. **(B)** Dendrogram constructed based on the correlation of gene expressions. **(C)** Heatmap of the correlation between modules and prognostic traits (Deeper colors indicate higher correlation, with red representing a positive correlation and blue representing a negative correlation). **(D)** Venn diagram of genes in the “Indianred” module and differentially expressed genes.

In addition, WGCNA was used to construct a gene co-expression network using the aforementioned dataset, and modules that correlated with the clinical features of heart failure were identified based on statistical differences. The similarity matrix was transformed into an adjacency matrix using a soft-threshold power of 9 (Figures 2A,B). Subsequently, the “Indianred” module showed a correlation coefficient of 0.4 with the clinical phenotype, indicating a significant statistical difference (Figure 2C).

**Figure 3:**
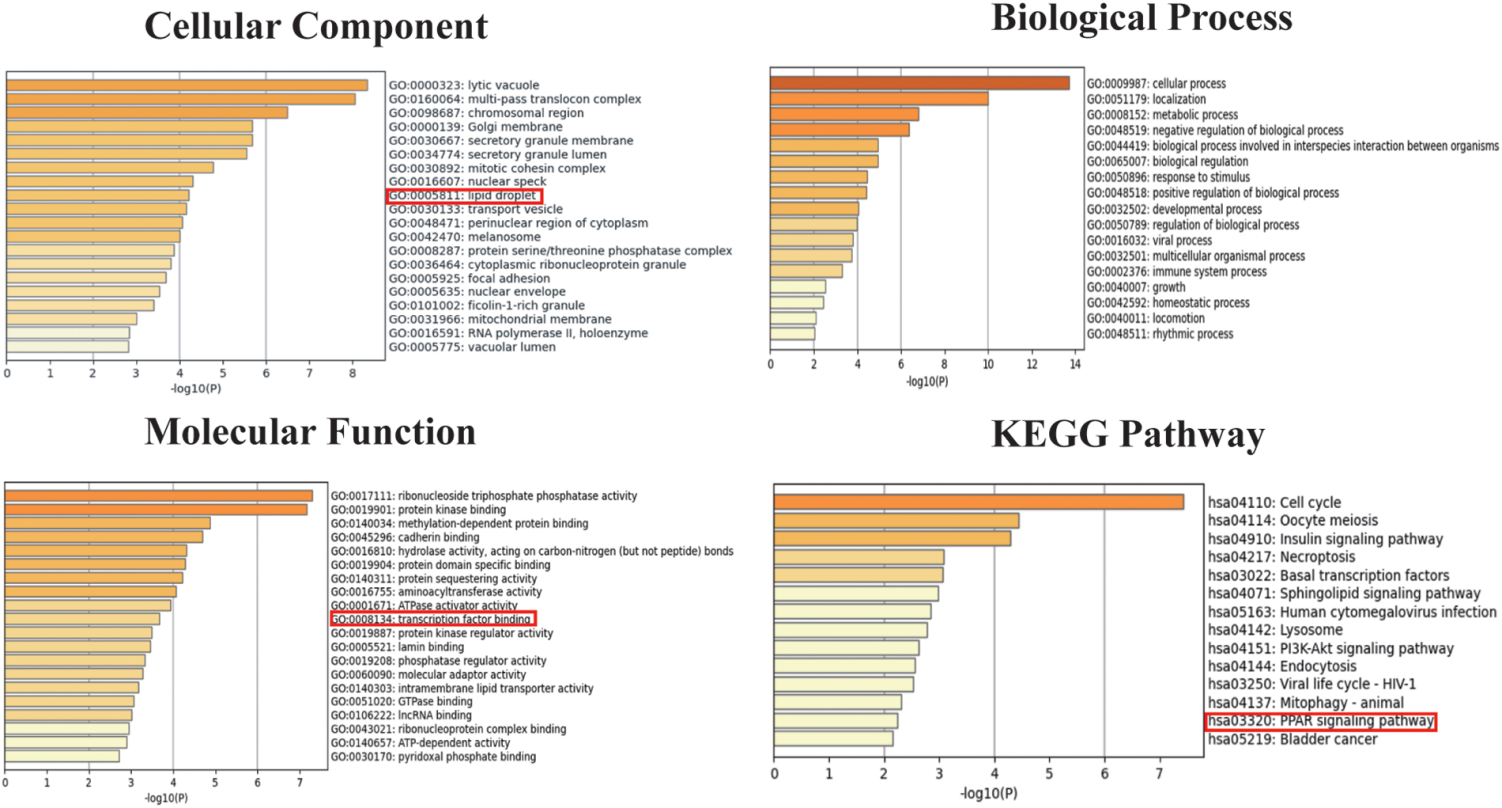
Go and KEGG enrichment analyses for both the differentially expressed genes and the genes in the “Indianred” module.

We selected 749 differentially expressed genes and intersected them with 1,600 genes from the “Indianred” module, resulting in a final list of 235 genes (Figure 2D). The top 20 genes are listed in order based on the absolute value of their fold change in Table 1. Comprehensive functional enrichment analyses, including GO and KEGG, were performed to elucidate the biological processes, pathways, molecular functions, and cellular components associated with the list of differentially expressed genes. Enrichment analysis revealed that the lipid-associated metabolic process was closely associated with the occurrence of HF including lipid droplets (PLIN2, ACSL4, RAB7A, RAB3GAP1, RAB18, SCCPDH, HSD17B11, and RAB10 were enriched) in the cellular component, PPAR signaling (PLIN2, ACSL1, ACSL4, ILK, and ELOVL5 were enriched) in the pathway component, and transcriptional factor binding (CDKN2A, DDX3X, NR3C1, GTF2I, HIF1A, HMGB2, NAB1, PRKDC, PSMD10, RAD21, TAF7, HIRA, UBE2I, YWHAZ, SUZ12, and MED30 were enriched) in the molecular function component (Figure 3).

**Table 1:** The top 20 genes differentially expressed genes displayed based on fold change.

Gene	Log_2_FC	*p*-value	Gene	Log_2_FC	*p*-value
VNN1	1.1488302	0.006351231	ACSL1	0.726556966	0.009636247
FLJ00290	−0.972567424	0.000368514	FACL2	0.726556966	0.009636247
PRKAR1A	0.964077515	0.000253927	RPS15	−0.723145345	0.041105412
SDCBP	0.939692912	0.00792319	FLJ36198	−0.722898397	0.009611904
SAMSN1	0.814208618	0.00191884	ZNF281	0.71855188	0.001850635
IFRD1	0.792488037	0.005234391	LOC90355	0.697261841	0.001967843
PSAP	0.789749715	0.001283008	STAG2	0.69271875	0.01945684
CGI-49	0.773432617	0.000772282	ACSL4	0.685246501	0.002850867
PRKDC	0.769715576	0.001105356	BTBD1	0.680257568	0.004046035
RIPK3	−0.726584269	0.005466932	GUK1	−0.669402303	0.012787984

### 3.2 Screening of LDRGs by machine learning for prognostic biomarkers

**Figure 4:**
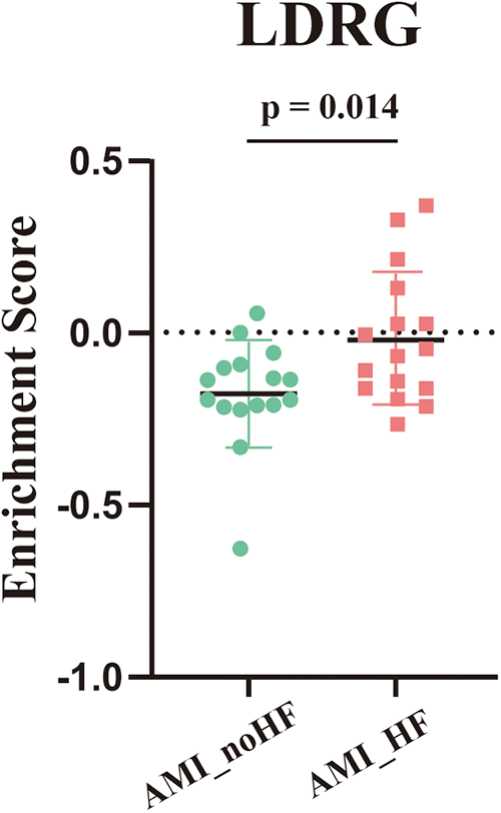
ssGSEA enrichment scores for the lipid droplet-associated genes across the samples in GSE11947 (*n* = 16, each group).

To further confirm whether the tendency to develop heart failure after an MI was strongly associated with LDRGs, we performed ssGSEA scoring of lipid droplet-related genes in the 32 samples from GSE11947. The result showed that the scores in the group that developed heart failure were significantly higher than those in the group that did not develop heart failure (Figure 4).

**Figure 5:**
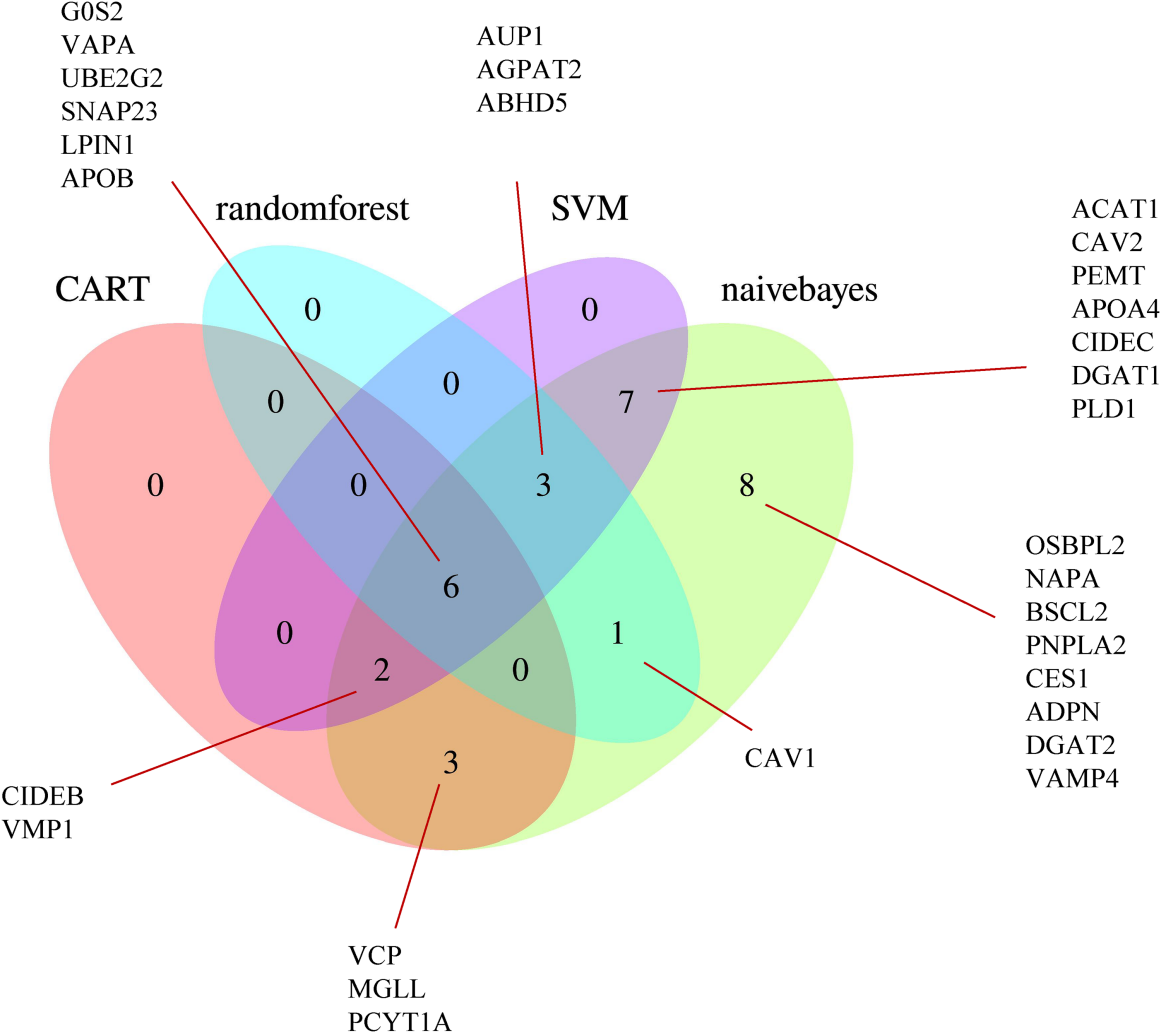
Venn diagram of the LDRGs identified by the CART, random forest, support vector machine, and naive Bayes machine-learning algorithms. G0S2, G0/G1 switch 2; VAPA, VAMP-associated protein A; UBE2G2, ubiquitin conjugating enzyme E2 G2; SNAP23, synaptosome-associated protein 23; LPIN1, lipin 1; APOB, apolipoprotein B; OSBPL2, oxysterol-binding protein-like 2; NAPA, NSF attachment protein alpha; BSCL2, Berardinelli-Seip congenital lipodystrophy 2; CAV1, caveolin 1; PNPLA2, patatin-like phospholipase domain-containing 2; CES1, carboxylesterase 1; ADPN, adiponectin; DGAT2, diacylglycerol O-acyltransferase 2; VAMP4, vesicle-associated membrane protein 4; AUP1, ancient ubiquitous protein 1; AGPAT2, 1-acylglycerol-3-phosphate O-acyltransferase 2; ABHD5, abhydrolase domain-containing 5; ACAT1, acetyl-CoA acetyltransferase 1; CAV2, caveolin 2; PEMT, phosphatidylethanolamine N-methyltransferase; APOA4, apolipoprotein A-IV; CIDEC, cell death-inducing DFFA-like effector C; DGAT1, diacylglycerol O-acyltransferase 1; PLD1, phospholipase D1; CIDEB, cell death-inducing DFFA-like effector B; VMP1, vacuole membrane protein 1; VCP, valosin-containing protein; MGLL, monoglyceride lipase; PCYT1A, phosphate cytidylyltransferase 1, choline, alpha.

Using the SVM, naive Bayes, CART, and random forest algorithms, we performed trained machine-learning models using the GSE11947 samples. After unifying the results, we identified 22 genes that were screened by at least two different machine-learning methods (Figure 5), namely, ABHD5, ACAT1, AGPAT2, APOA4, AUP1, CAV2, CIDEB, CIDEC, DGAT1, G0S2, LPIN1, MGLL, PCYT1A, PEMT, PLD1, SNAP23, UBE2G2, VAPA, VCP, VMP1, CAV1, and APOB.

### 3.3 Construction of the TF-LDRG relationship network

**Figure 6:**
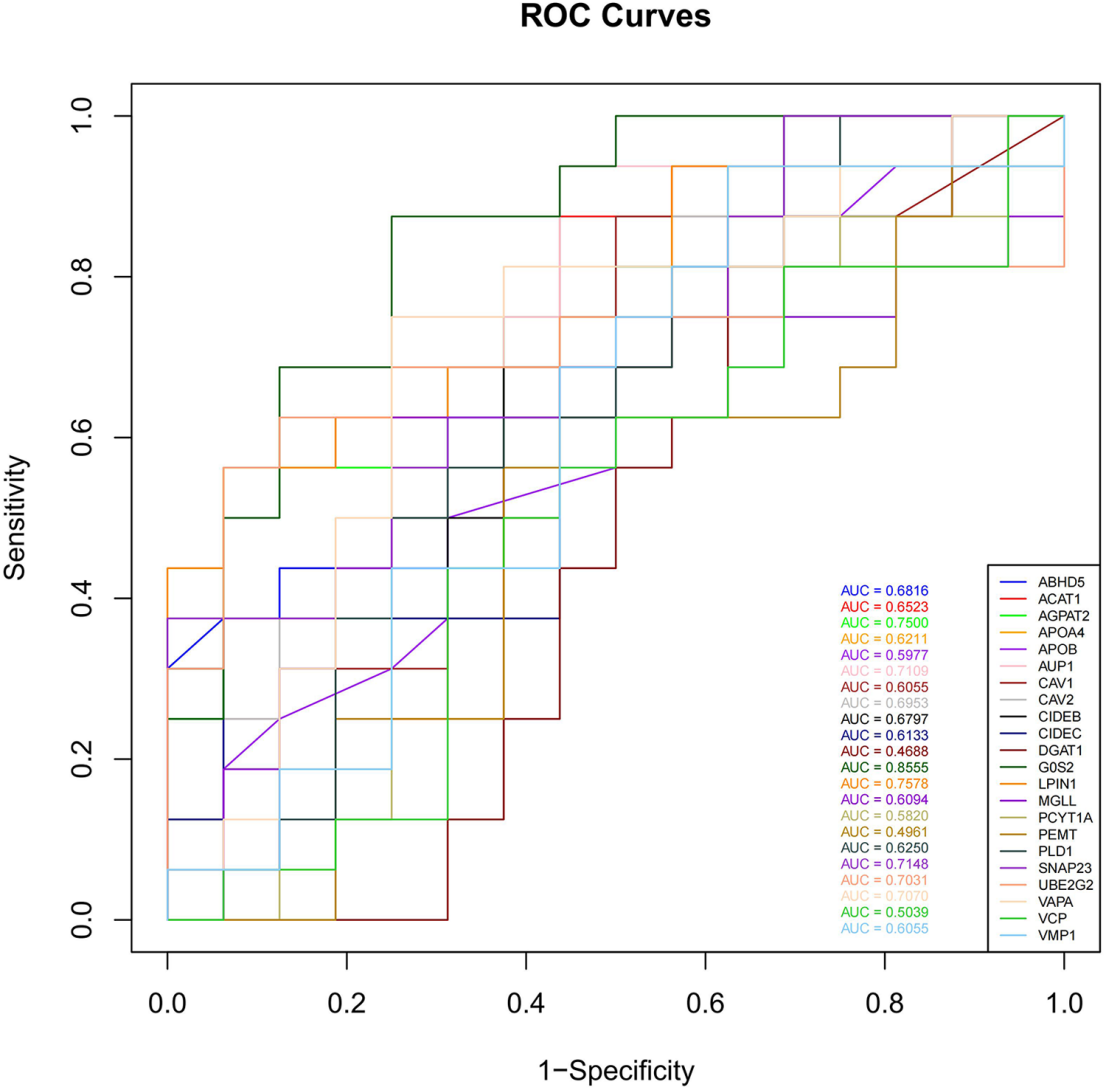
The ROC curves of 22 LDRGs for heart failure classification in the GSE11947 microarray data.

To assess the predictive ability of the selected LDRGs for heart failure after an AMI, we analyzed the expression of LDRGs in the non-heart failure and heart failure groups in the GSE11947 dataset and constructed ROC curves for each LDRG. The AUC indicates the specificity and sensitivity of the LDRG for predicting heart failure. By constructing ROC curves, each LDRG was assessed for its prediction of the occurrence of heart failure. It was found that only the G0S2 gene had an AUC (AUC = 0.8555) greater than 0.8. The AUC values for the other genes are shown in Figure 6.

**Figure 7:**
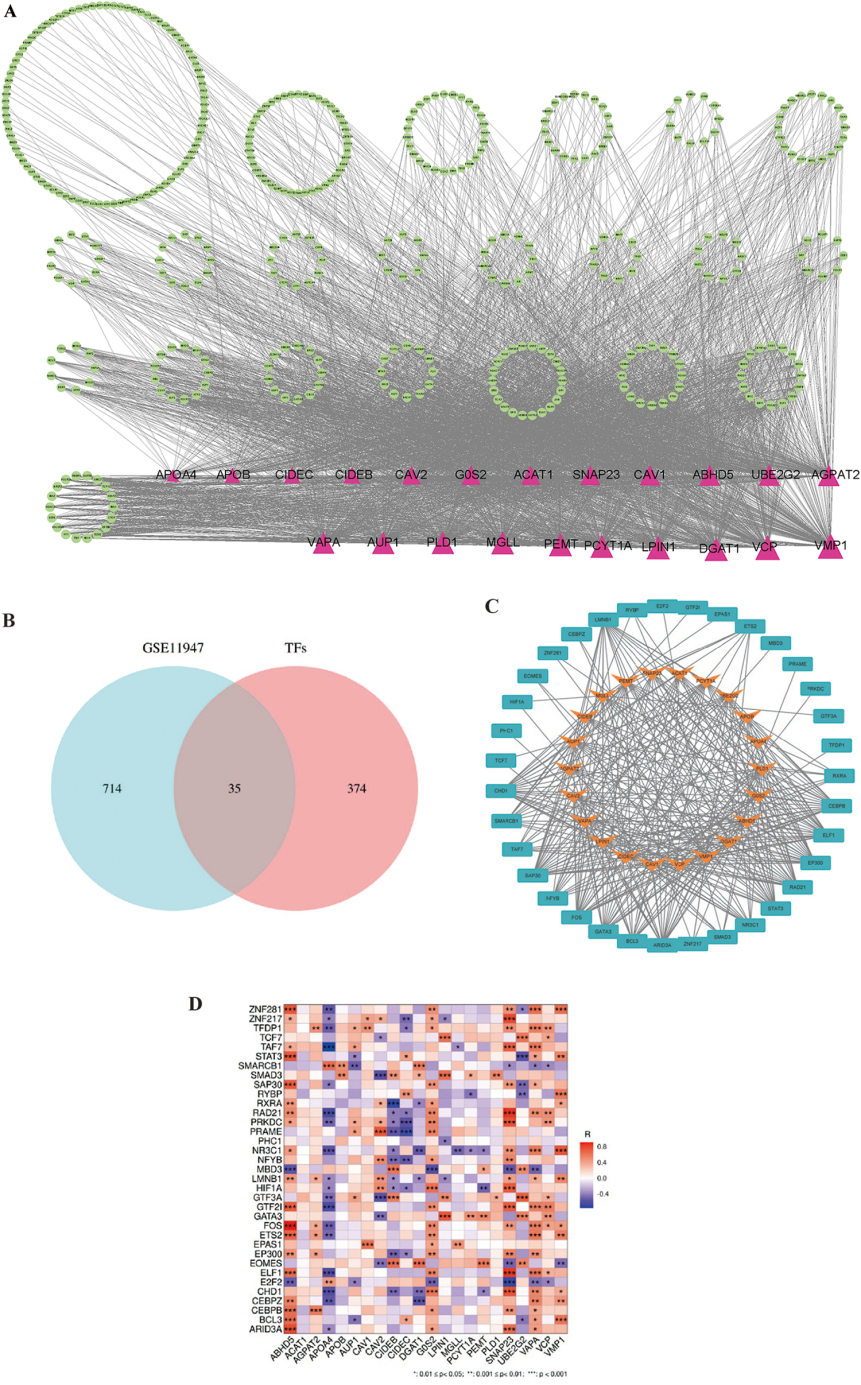
The transcription factor and lipid droplet-related gene expression network construct. **(A)** The overall PPI network with 22 transcriptional factors and 409 lipid droplet-related genes. Triangles represent LDRGs. TFs are represented by circles. The lines connecting TFs and LDRGs indicate an LDRG that is predicted to be regulated by a TF. **(B)** Venn diagram of the predicted transcription factors and differentially expressed genes. **(C)** PPI network of the transcription factors and lipid droplet-related genes. **(D)** Correlation heatmap for 35 transcription factors and 22 lipid droplet genes.

Transcription factors help us to understand the regulatory mechanisms controlling LDRG expression, which can provide insights into their roles and potentially predict the prognosis in AMI-induced HF. A search for 22 lipid droplet-related genes was conducted in the ChEA, ENCODE, hTFtarget, TRANSFAC, and TRRUST databases, which resulted in 409 relevant transcription factors being identified (Figure 7A). In these databases, a cohort of 35 TFs was predicted that showed differential expression between the non-HF and HF groups (Figure 7B). The STRING database was then used to generate an illustrative visualization of the associations between the TFs and LDRGs in the PPI network (Figure 7C). A correlation heatmap was created to highlight the expression relationships between the TFs and LDRGs (Figure 7D).

### 3.4 Evaluation of TF-LDRG prognostic biomarkers in predicting HF after a myocardial infarction

**Figure 8:**
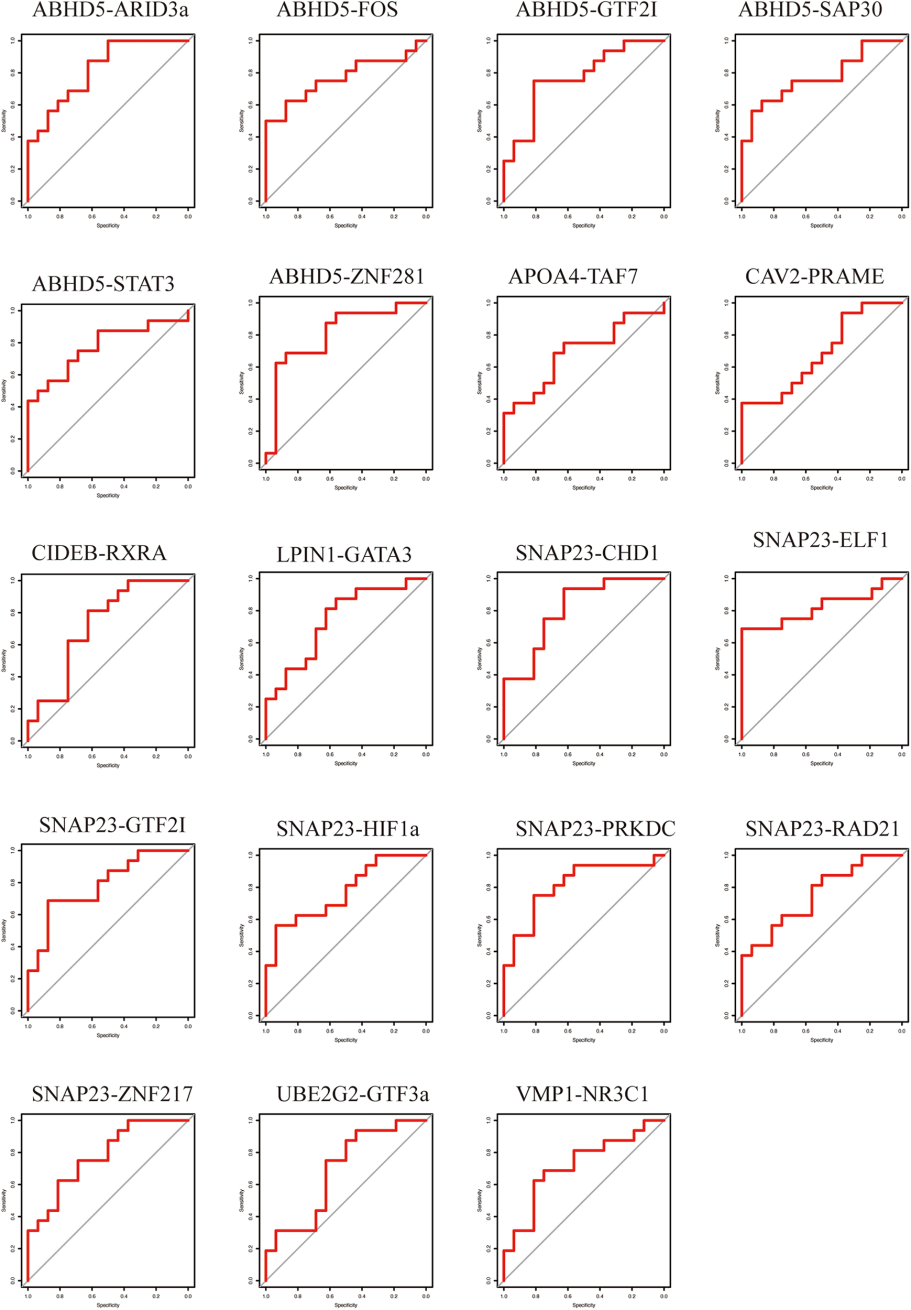
The ROC curve of the TF-LDRG classification effect in the GSE11947 microarray dataset.

To evaluate the predictive ability of the TF-LDRGs for HF post-AMI, the combined expression of TFs and LDRGs in the non-HF and HF samples from the GSE11947 dataset were analyzed and ROC curves were constructed. The AUC, demonstrating the specificity and sensitivity of the system, tested the validity of the test. The ability of a single LDRG to predict the occurrence of HF was evaluated by constructing the ROC curves. The AUC was calculated for each of them and only G0S2 (AUC = 0.8555) had an AUC greater than 0.8. Therefore, we could potentially apply the TF-LDRG system to increase the efficacy of HF prediction. The expression correlation between the TFs and LDRGs was abs(*R*) >0.7 and *p* <0.01. The prognostic value of different combinations of TF-LDRGs, namely, ABHD5-ARID3a (AUC = 0.8203), ABHD5-ZNF281 (AUC = 0.8086), SNAP23-CHD1 (AUC = 0.8086), SNAP23-ELF1 (AUC = 0.8203), and SNAP23-PRKDC (0.8125), had AUCs greater than 0.80, indicating good performance in predicting HF status after a myocardial infarction and a better performance than that of a single LDRG (Figure 8).

### 3.5 Validation of the TF-LDRG prognostic biomarkers in predicting HF after a myocardial infarction

**Figure 9:**
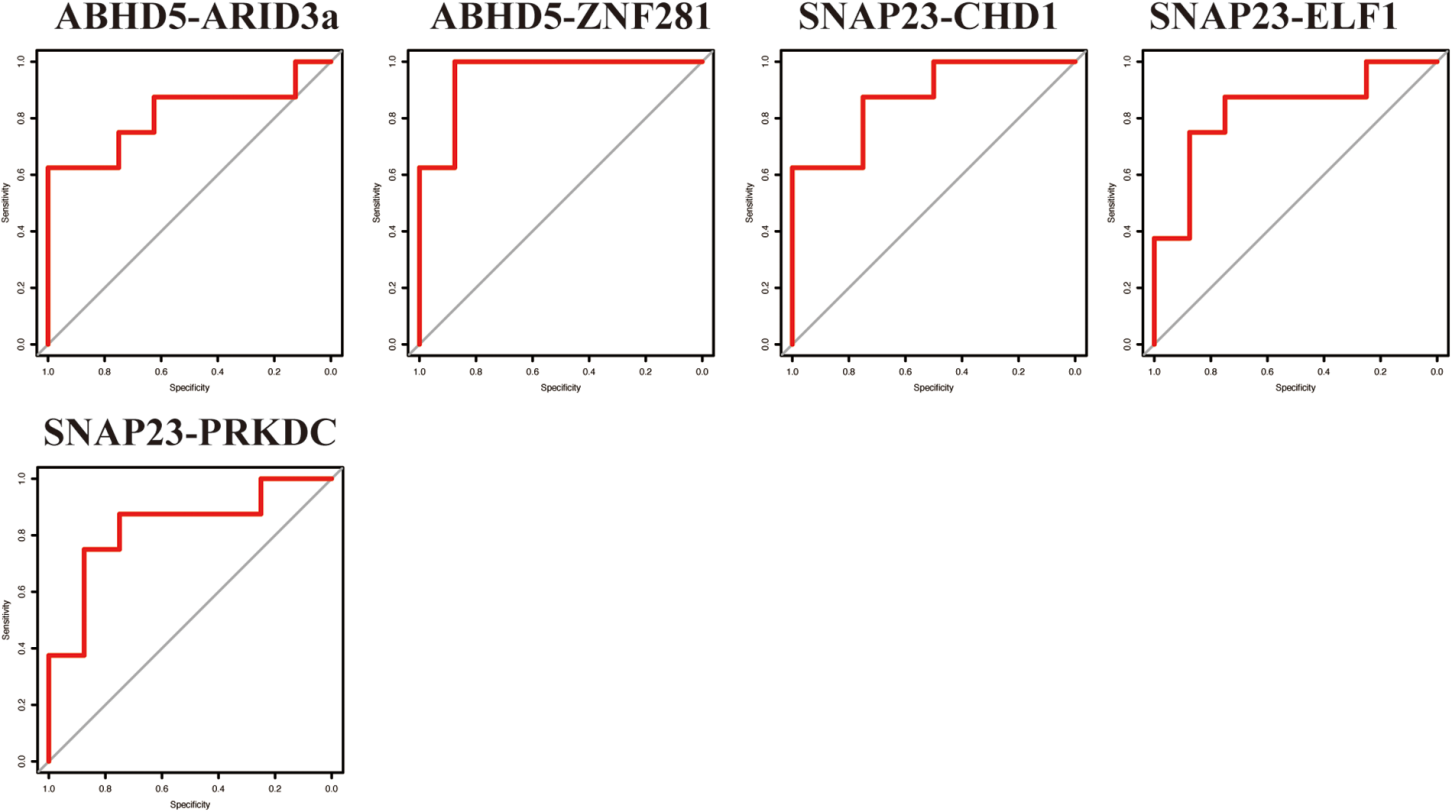
The ROC curve of the TF-LDRG classification effect in the GSE55897 microarray dataset for validation.

To verify the results from the GSE11947 dataset, these TF-LDRG expression combinations were validated using GSE55897. ROC curves were constructed to evaluate the selected TF-LDRG combinations. The results showed that the combination of ABHD5-ARID3a (AUC = 0.8125), ABHD-ZNF281 (AUC = 0.9531), SNAP23-CHD1 (AUC = 0.875), SNAP23-ELF1 (AUC = 0.8281), and SNAP23-PRKDC (0.8281) had AUCs greater than 0.80 (Figure 9).

**Figure 10:**
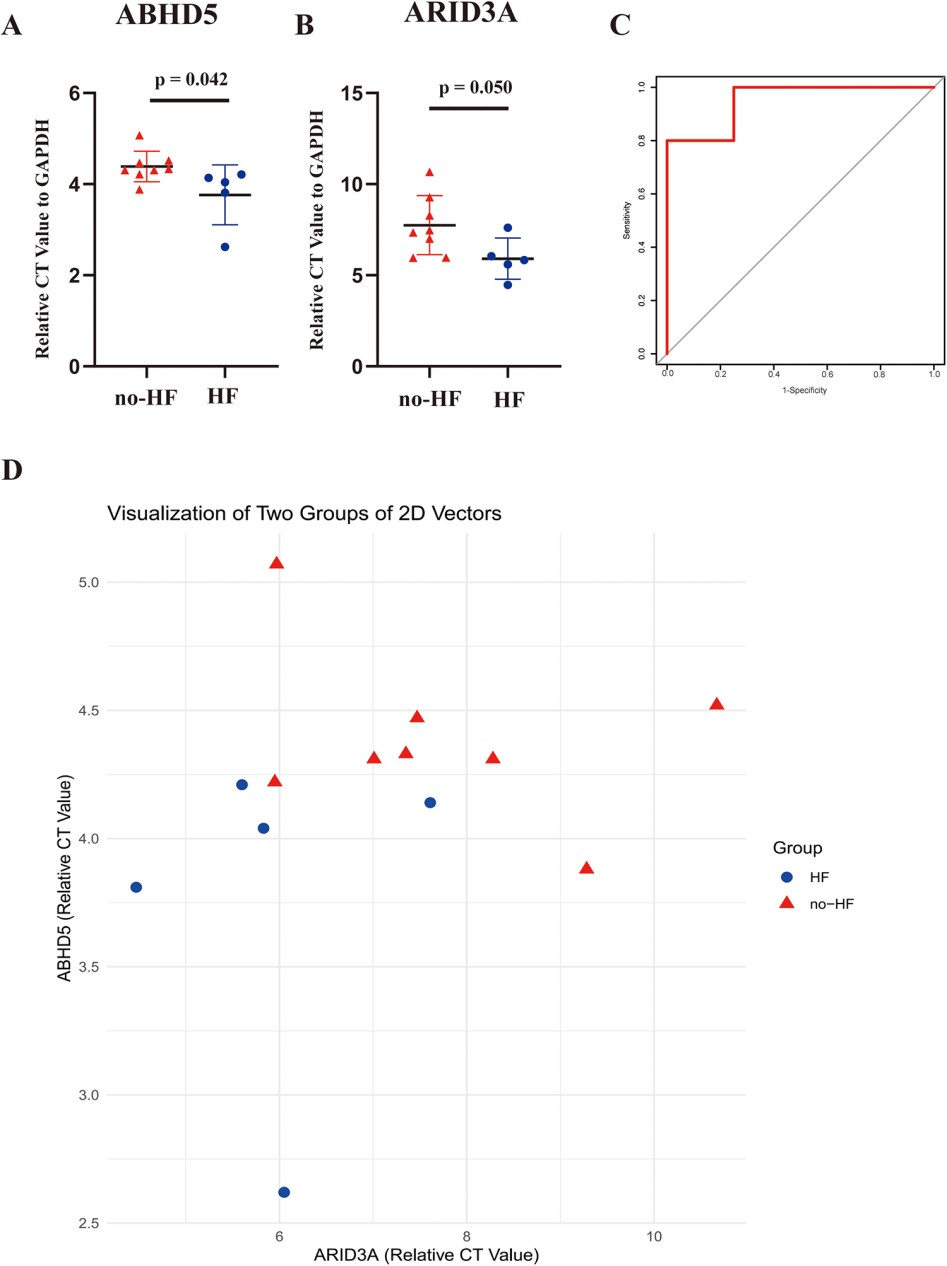
Validation of the TF-LDRG prognostic biomarkers in predicting HF in clinical samples. **(A,B)** The relative CT values of ABHD5 and ARID3a compared to GAPDH in peripheral blood samples in the non-heart failure and heart failure groups. **(C)** The ROC curve of ABHD5-ARID3a gene expression in the peripheral blood samples for predicting heart failure. **(D)** Two-dimensional scatter plots of the relative CT values of the TF-LDRG gene pairs compared to GAPDH in the non-heart failure and heart failure groups.

Subsequent validation was conducted using the peripheral blood obtained from clinical specimens, with detailed patient information available in Table 2. The results showed that the relative cycle threshold (CT) value of ABHD5 was significantly lower in the AMI_HF group than in the AMI_noHF group (Figure 10A). A similar tendency was also found in terms of ARID3a in the peripheral blood samples (Figure 10B). The AUC result for ABHD5-ARID3a in predicting heart failure was 0.95, which showed good performance (Figure 10C). The two-dimensional vectors between the groups indicated a pronounced disparity in the expression levels of the gene pair ABHD5-ARID3a (Figure 10D). Specifically, it was observed that in the peripheral blood of patients who developed heart failure following a myocardial infarction, there was an elevation in the relative CT values, manifesting as an increased expression in ABHD5-ARID3a.

**Table 2:** Baseline demographic and clinical characteristics of the non-HF and HF patients from Jiangsu Province Hospital of Integration of Chinese and Western Medicine.

Characteristic	Non-HF patients (*n* = 8)	HF patients (*n* = 5)	*p*-value
Sex (male)	6 (75%)	3 (60%)	>0.999
Age (years)	58.0 ± 11.6	65.8 ± 10.6	0.286
Smoking	5 (62.5%)	0 (0%)	0.075
Hypertension	2 (25%)	3 (60%)	0.293
Diabetes	1 (12.5%)	2 (40%)	0.511
Dyslipidemia	3 (37.5%)	0 (0%)	0.231
Previous MI	0 (0%)	1 (20%)	0.385

Data were presented as mean value ± standard deviation or percentage of patients.

## 4 Discussion

Myocardial infarction causes great damage to the myocardium, leading to cardiac dysfunction and even heart failure (22). Strategies to protect cardiomyocytes and prevent progression to heart failure have become a concern in the medical field. Therefore, accurately predicting the probability of patients progressing to HF after an MI is crucial in reducing the overall incidence of HF.

**Figure 11:**
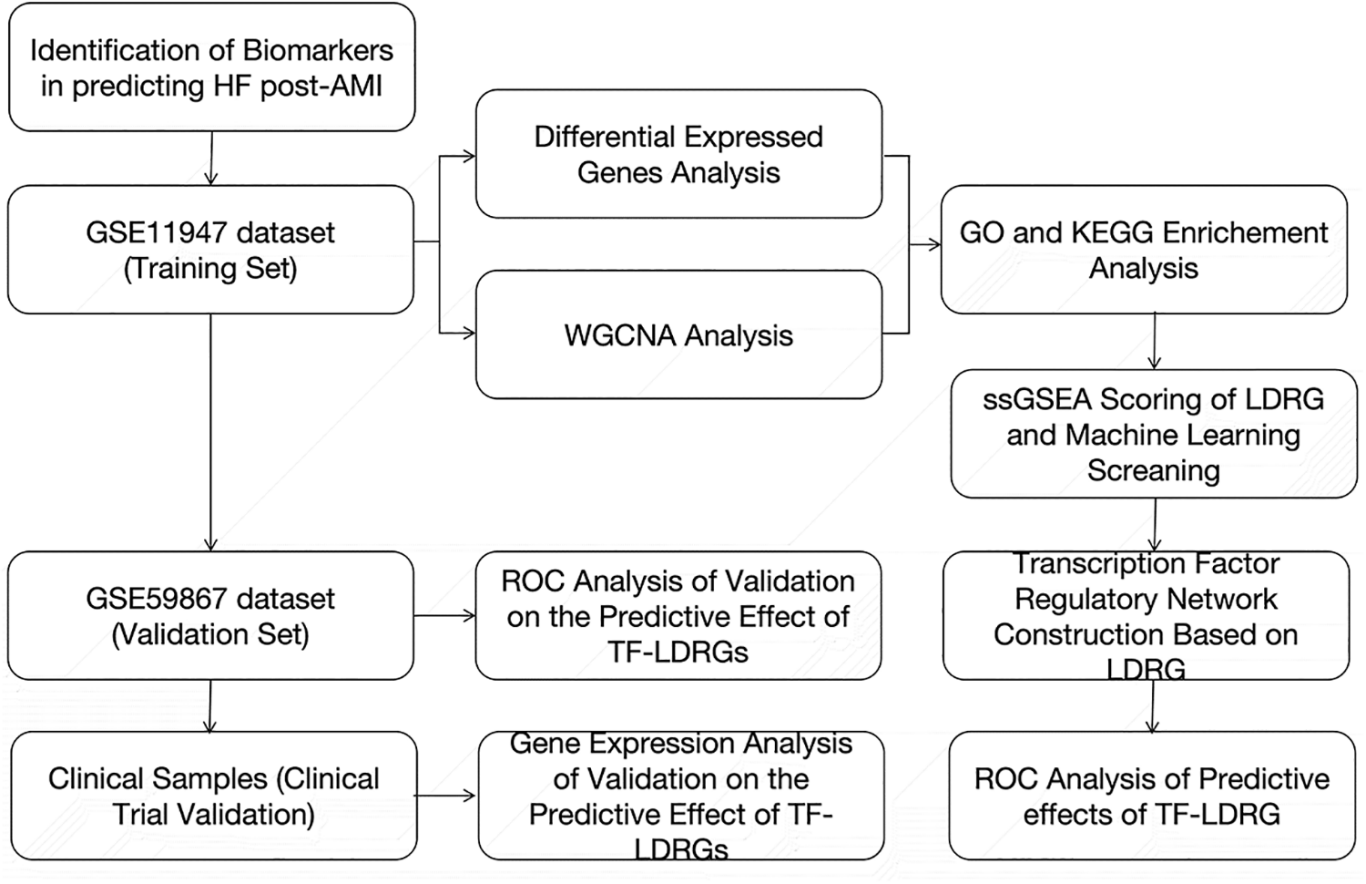
The flow diagram of the whole study.

The widespread adoption of high-throughput transcriptomic technologies and the reduction in their associated costs have expanded the feasibility of large-scale investigations into the gene expression levels in tissues. This advancement also offers the possibility of predicting HF in patients with an AMI by analyzing the gene expression profiles in their peripheral blood, leading to the identification of individuals at high risk of developing HF and early interventions to reduce the risk of progression to HF. In this study, we collected the clinical samples from the GSE11947 dataset, which includes the whole blood cell gene expression profiles of patients within 12 h after the onset of an AMI. The patients were divided into two groups based on whether they progressed to HF within 1 month. Using the “limma” package, differentially expressed genes were selected. Subsequently, the WGCNA method was employed to identify modules correlated with prognostic phenotypes, and the genes within this module, which intersected with the differentially expressed genes, were found to be enriched in fatty acid metabolism and lipid droplet-associated proteins. Various machine-learning models were then utilized to filter out 22 key lipid droplet-related genes, and databases were used to predict the upstream TFs of these 22 genes. We identified five co-expressed TF-LDRG pairs that appeared to have promising prognostic predictive value in the testing dataset and, of these, ABHD5-ARID3a was subsequently validated to have good performance in predicting HF in the clinical trial (Figure 11).

After an AMI, myocyte energy metabolism is disrupted, with a shift in energy use from fatty acid oxidation to glycolysis under ischemic and hypoxic conditions (23). Even after revascularization, mitochondrial dysfunction persists due to calcium overload and a surge of oxygen radicals, leading to impaired nutrient metabolism (24). In this study, functional enrichment analysis was performed on the HF-related module (the “Indianred” module identified by WGCNA) and differentially expressed genes, which were enriched in the metabolism-related molecular components and physiological processes such as PPAR signaling, PI3K signaling, ATP generation, and lipid droplet metabolism, revealing that these core genes are closely related to lipid homeostasis and metabolic reprogramming (25, 26). Starting with lipid droplet-related genes closely related to lipid metabolism, and utilizing techniques such as machine learning, an attempt was made to find TF-LDRG combinations with good prognostic predictive effects, providing a new direction for exploring biomarkers for early identification of AMI patients at high risk for HF progression.

Recent studies have demonstrated that the elevation in inflammatory levels following an MI can accelerate the progression of metabolic dysfunction-associated steatohepatitis (MASH) (27). This evidence suggests that a myocardial infarction not only causes damage to the heart but also acts as a systemic disease affecting multiple organs throughout the body. The metabolites, myocardial enzymes, and inflammatory responses that follow cardiac injury influence the metabolism of peripheral blood cells, including lipid and energy metabolism (28). In the presence of oxidative stress, monocytes inhibit the function of HMG-CoA reductase (HMGCR) and Rac1 prenylation, which subsequently reduces the uptake, transport, and utilization of lipids by the cells (29). Conversely, alterations in lipid metabolism in peripheral blood cells have the potential to impact cardiac function prognosis. For example, alterations in monocyte lipid metabolism directly impact the function of vascular endothelial cells in the heart, influencing vascular tone, inflammatory responses, and thrombosis, all of which are crucial for cardiac function (30). The impact of PLIN2 on local atherosclerotic lipid load and inflammation may also contribute to the exacerbation of the no-reflow phenomenon following a primary PCI in STEMI patients (31). However, the current evidence linking lipid metabolism in peripheral blood cells to heart failure remains insufficient. This study aimed to provide a direction for further exploration of the intrinsic relationship between the two by conducting data analysis and preliminary validation.

The genes we screened out have been implicated in previous studies as potentially relevant to the development of HF. HF is often accompanied by cell injury, death, and phenotypic changes. AT-rich interactive domain-containing protein 3a (ARID3a) is involved in DNA binding and gene expression regulation and is particularly associated with chromatin remodeling, gene transcription activation, and immune regulation (32). ARID3a regulates cell proliferation, differentiation, and repair (33). Cardiac fibrosis after a myocardial infarction is mainly associated with cardiac fibroblasts, and their differentiation is the key pathological process closely related to the progression of HF. A study showed that reduced levels of ARID3a forced fibroblast-to-myofibroblast differentiation of TGF-β-induced human cardiac fibroblasts (HCFs). Therefore, ARID3a may be a novel therapeutic target for cardiac fibrosis and post-MI HF (34). α/β-hydrolase domain-containing protein (ABHD)5 is a member of the ABHD protein family. It plays a critical role in lipid metabolism. ABHD5 interacts with perilipins on lipid droplets, thereby regulating lipolysis, the breakdown of fats into fatty acids and glycerol, which is essential for energy production and lipid homeostasis in cells (35). It has been verified that cardiac-specific ABHD5 deficiency activates endoplasmic reticulum stress to promote heart failure in mice (36). It has also been reported that ABHD5 proteolyzes HDAC4 to maintain cardiometabolic homeostasis during pressure overload and lipotoxicity-induced heart failure (37). Based on our results and previous reports, ABHD5-ARID3a could be a potential “watchdog” for HF post-AMI. The specific mechanisms by which these genes contribute to the progression of heart failure require further investigation.

Nonetheless, there are several limitations to this study. First, the sample size is modest, with the GSE11947 and GSE59867 datasets including a total of only 48 valid clinical samples, which is insufficient for training machine-learning models and leads to instability in the results. Second, microarray data are less sensitive than RNA-seq for detecting low-abundance transcripts, making it difficult to differentiate subtypes and identify gene variants (38). In these datasets, key data such as ejection fraction (EF) values or NT-proBNP levels were not available when the blood samples were taken 12 h after an AMI. Furthermore, if some HF patients had already reduced ejection fraction at admission, it could affect the accuracy of the screened genes as biomarkers for predicting HF after an AMI. Finally, our patient sample size was also small. We plan to continue to increase the number of patients in future studies to verify the efficacy of the screened TF-LDRG combinations in predicting heart failure.

## Data Availability

Publicly available datasets were analyzed in this study. This data can be found here: https://www.ncbi.nlm.nih.gov/geo, accession numbers GSE11947 and GSE59867.
